# Supplementary material for: Exploring the Relationship between Biosynthetic Gene Clusters and Constitutive Production of Mycosporine-like Amino Acids in Brazilian Cyanobacteria
Source: Molecules. 2023 Feb 2;28(3):1420. doi: 10.3390/molecules28031420 (PMC9918943; doi:10.3390/molecules28031420)
Supplement: Supplementary file 1 [file molecules-28-01420-s001.zip › molecules-2036267-supplementary.pdf]

Exploring the relationship between biosynthetic gene clusters and constitutive production of mycosporine-like amino acids in Brazilian cyanobacteria

Rafael B. Dextro

University of São Paulo (USP), Center for Nuclear Energy in Agriculture (CENA), Avenida Centenário 303, 13416-000 Piracicaba, SP, Brazil

Endrews Delbaje

University of São Paulo (USP), Center for Nuclear Energy in Agriculture (CENA), Avenida Centenário 303, 13416-000 Piracicaba, SP, Brazil

Vanessa Geraldes

University of São Paulo (USP), School of Pharmaceutical Sciences, Avenida Prof. Lineu Prestes 580, 05508-000, São Paulo, SP, Brazil

Ernani Pinto

University of São Paulo (USP), Center for Nuclear Energy in Agriculture (CENA), Avenida Centenário 303, 13416-000 Piracicaba, SP, Brazil

Paul F. Long

University of São Paulo (USP), School of Pharmaceutical Sciences, Avenida Prof. Lineu Prestes 580, 05508-000, São Paulo, SP, Brazil

Faculty of Life Sciences & Medicine, King's College London, United Kingdom

Marli F. Fiore

University of São Paulo (USP), Center for Nuclear Energy in Agriculture (CENA), Avenida Centenário 303, 13416-000 Piracicaba, SP, Brazil

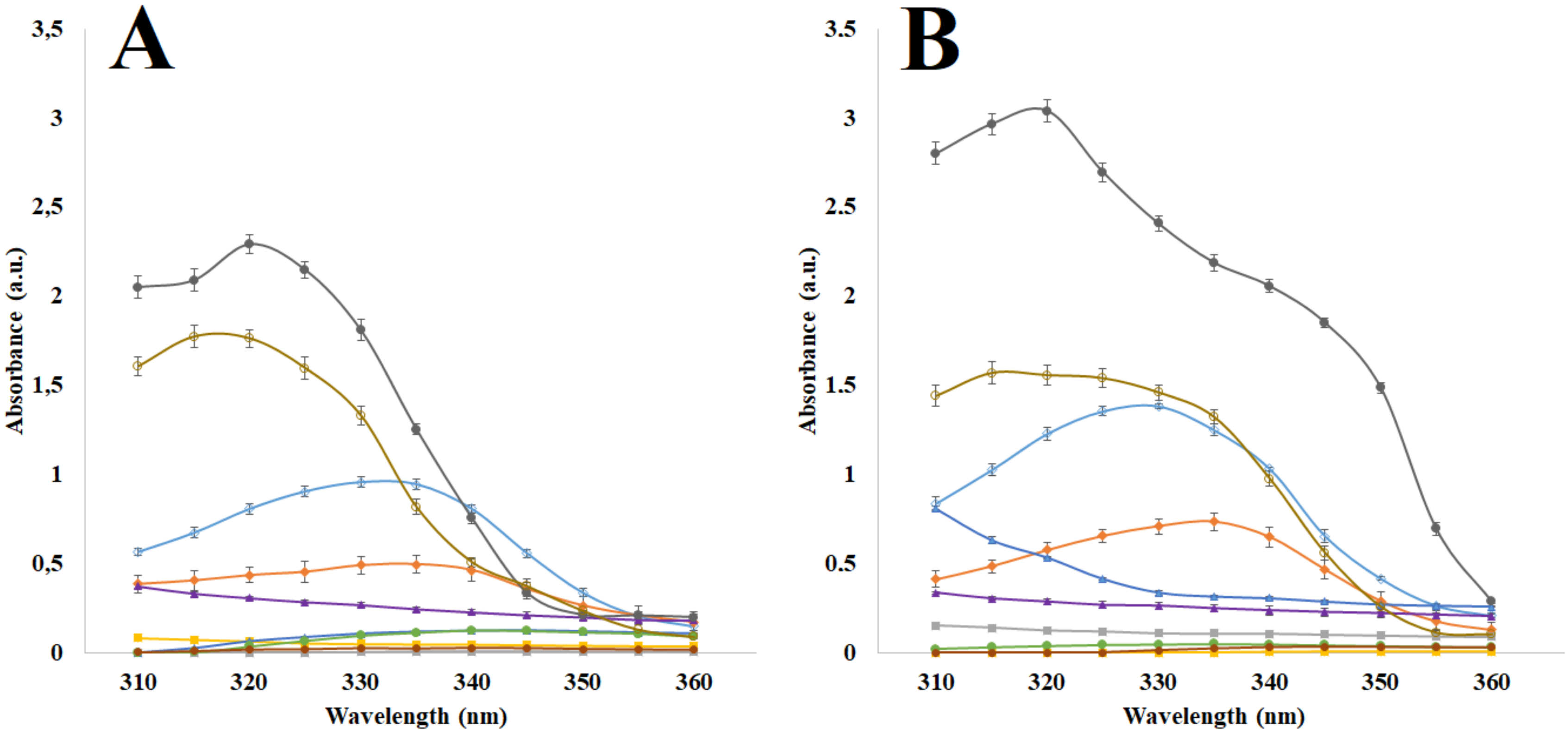

Figure S1. Absorbance detected on the wavelength of 310 to 360 nm at the cyanobacterial extracts cultivated at fluorescent light (A) and with UV stimulus (B). Strains tags: ● UFV-OR1; ○ CENA114; ◊ ITEP-024; ♦ CENA596; ● CENA516; ● CCIBt3563; ■ CENA597; ■ CENA650; ▲ CENA526; ▲CENA528.

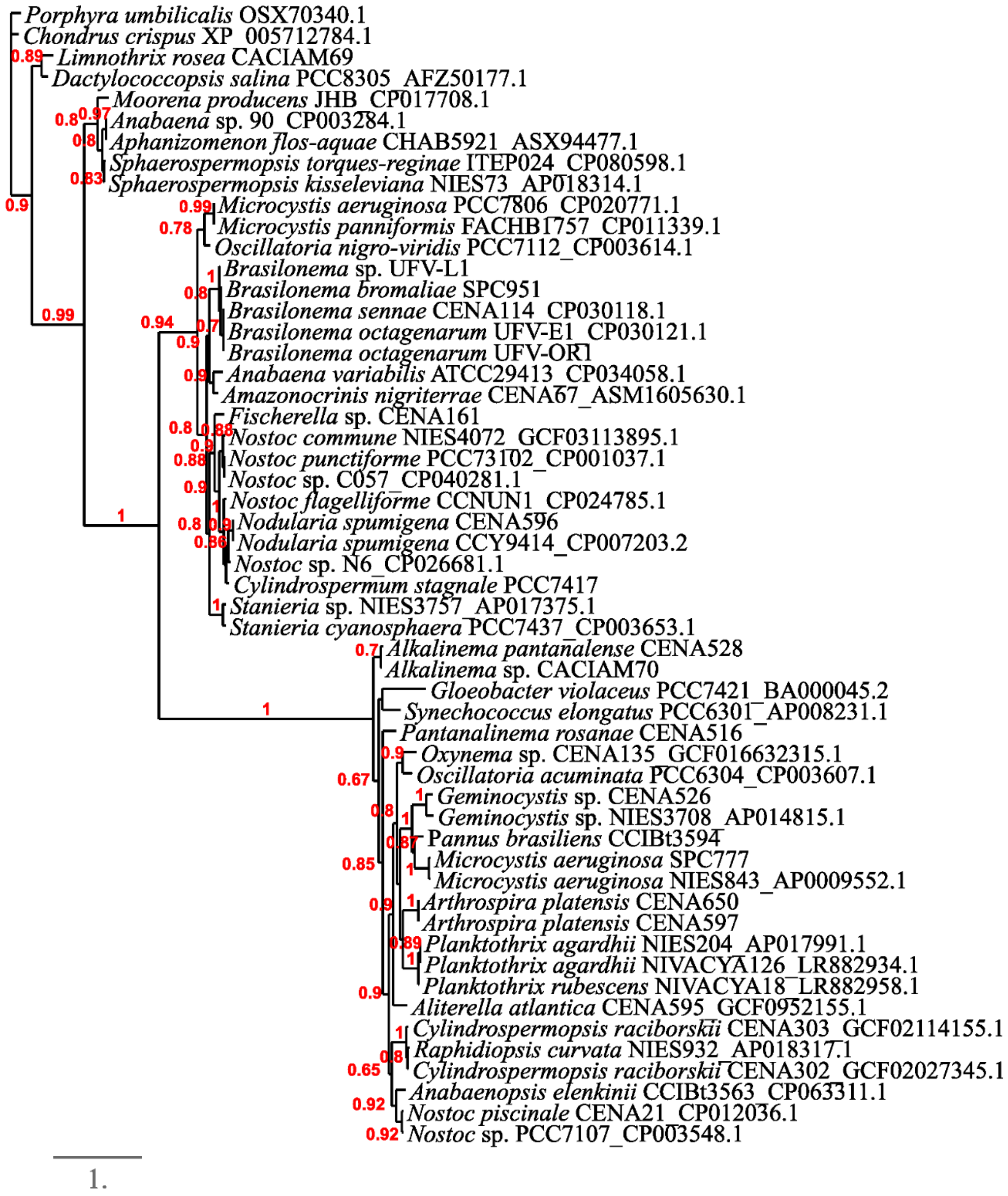

Figure S2. Maximum likelihood phylogeny tree of the gene encoding for 3-dehydroquinate synthase showing the evolutionary relationship of each gene's copy from cyanobacteria, other bacteria (*Dactylococcopsis salina* PCC8305) and red algae (*Porphyra umbilicalis* and *Chondrus crispus*)

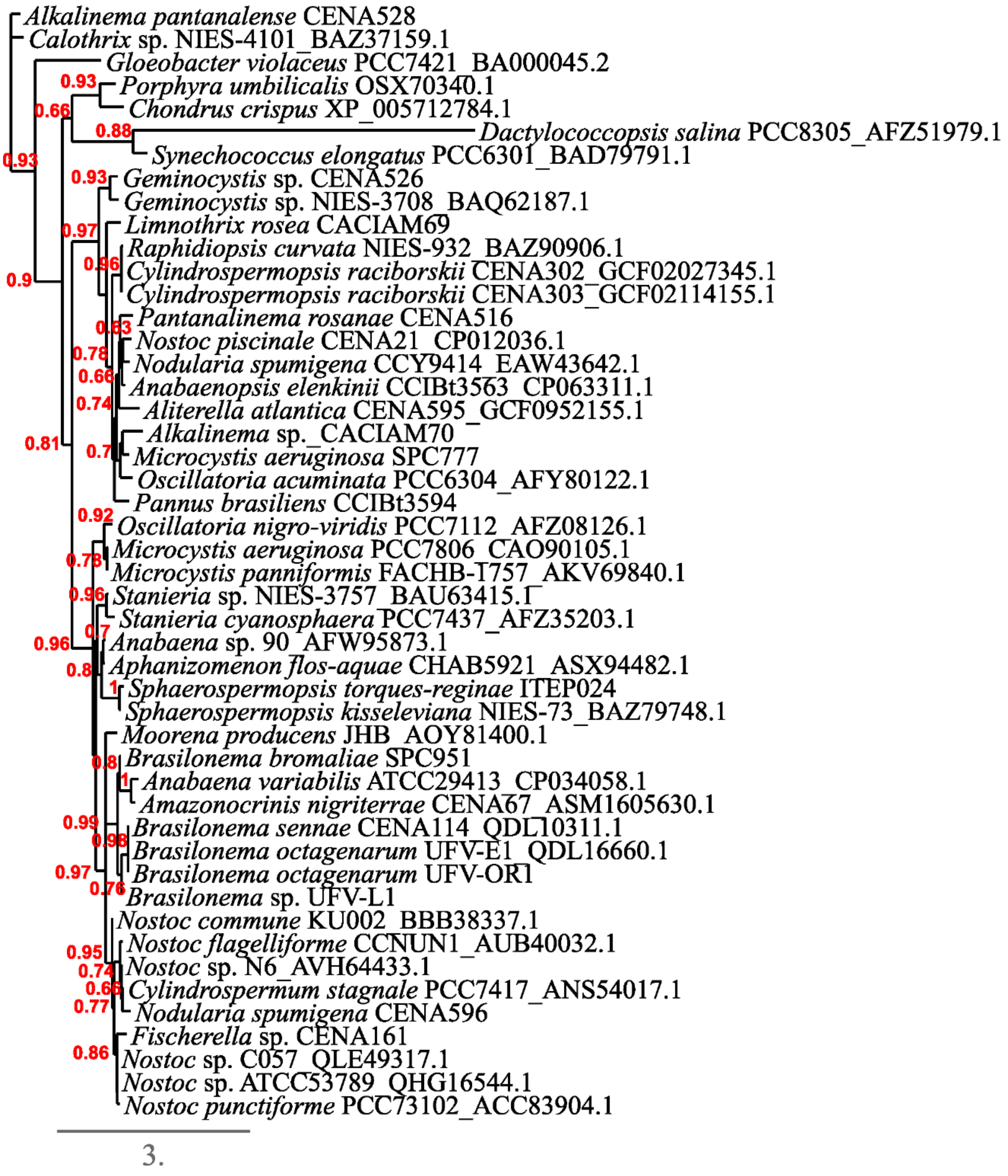

Figure S3. Maximum likelihood phylogeny tree of the gene encoding for *O*-methyltransferase showing the evolutionary relationship of each gene's copy from cyanobacteria, other bacteria (*Dactylococcopsis salina* PCC8305) and red algae (*Porphyra umbilicalis* and *Chondrus crispus*)

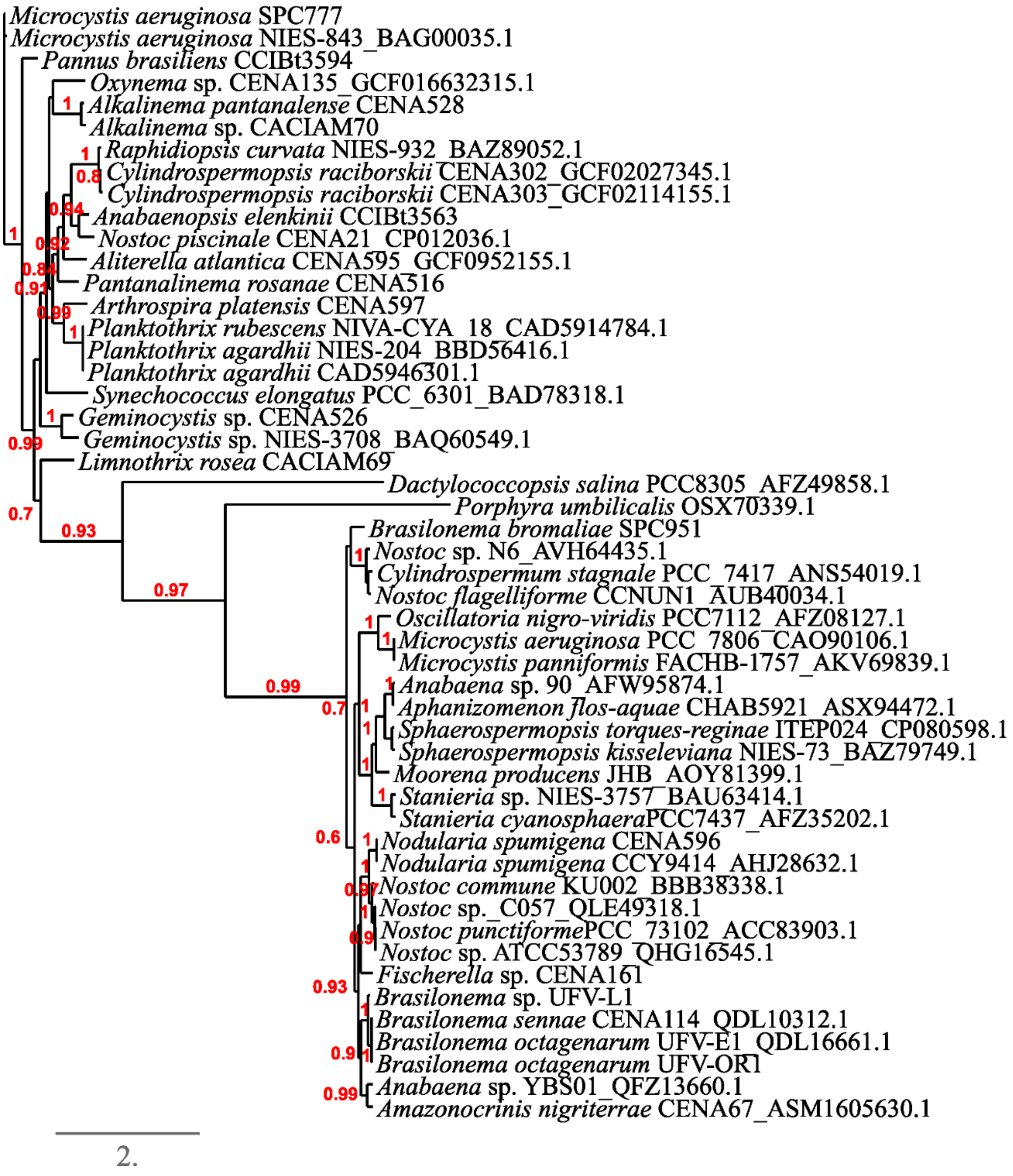

Figure S4. Maximum likelihood phylogeny tree of the gene encoding for ATP-grasp showing the evolutionary relationship of each gene's copy from cyanobacteria, other bacteria (*Dactylococcopsis salina* PCC8305) and red algae (*Porphyra umbilicalis*)

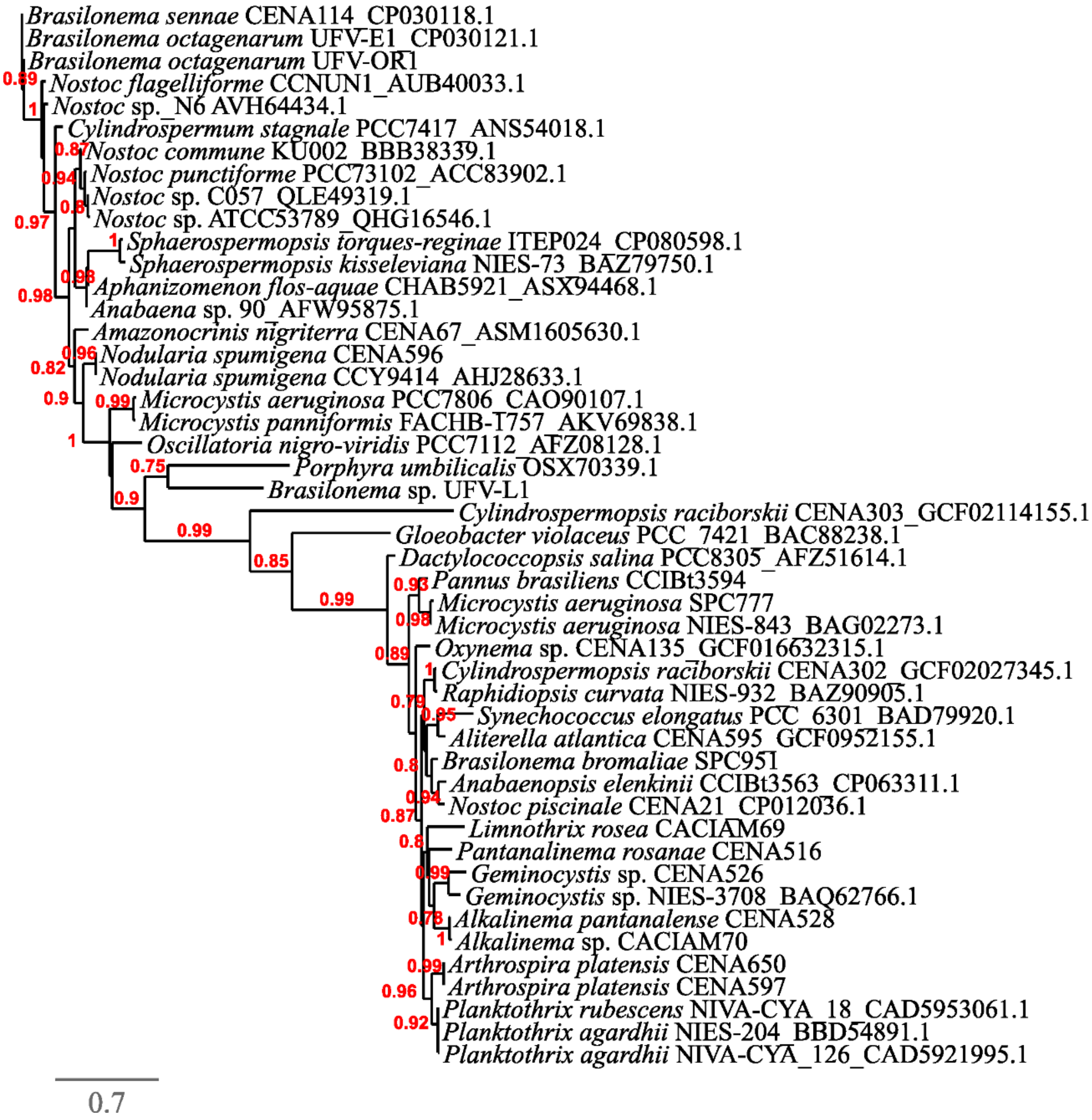

Figure S5. Maximum likelihood phylogeny tree of the gene encoding for d-Ala d-Ala ligase showing the evolutionary relationship of each gene's copy from cyanobacteria, other bacteria (*Dactylococcopsis salina* PCC8305) and red algae (*Porphyra umbilicalis*)

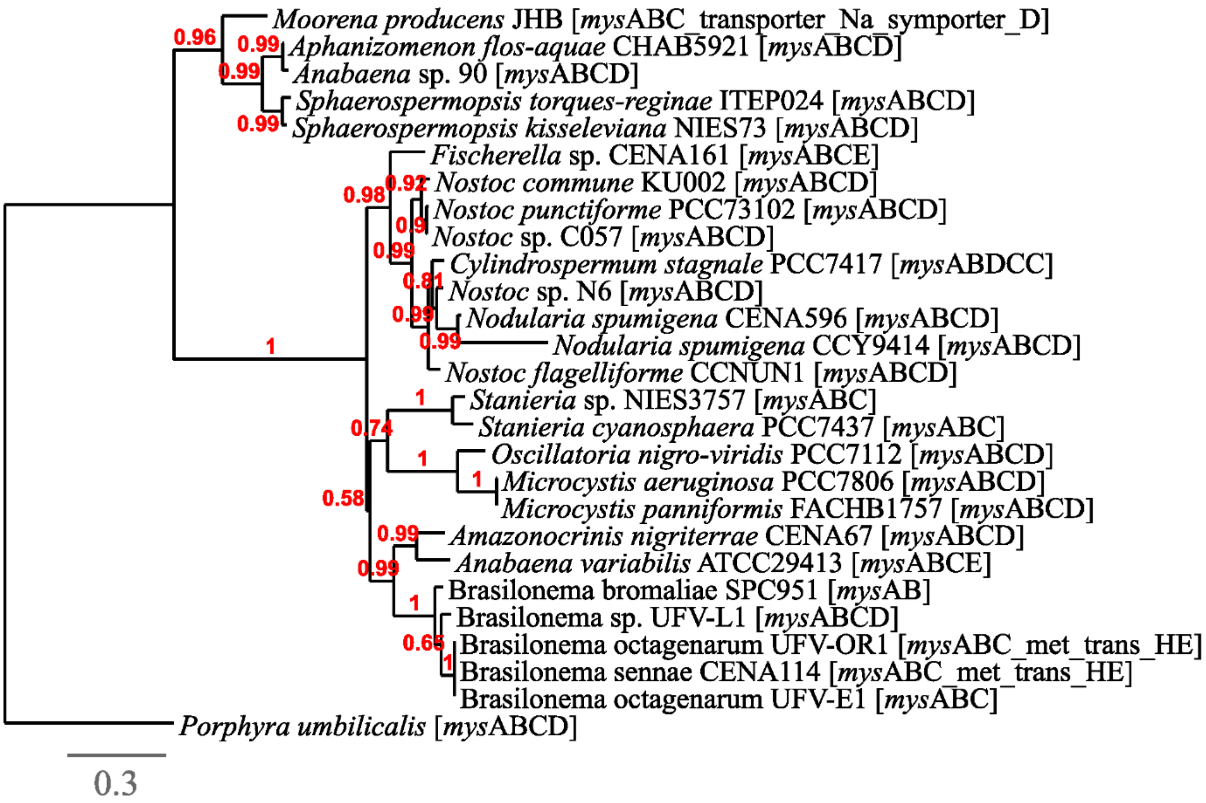

Figure S6. Maximum likelihood phylogeny tree of the MAA-related gene cluster found in the selected cyanobacterial strains that contained colinear copies of the *mys* genes and a red alga (*Porphyra umbilicalis*) showing the evolutionary relationship between each cluster. The gene composition of each strains' cluster is displayed between brackets.

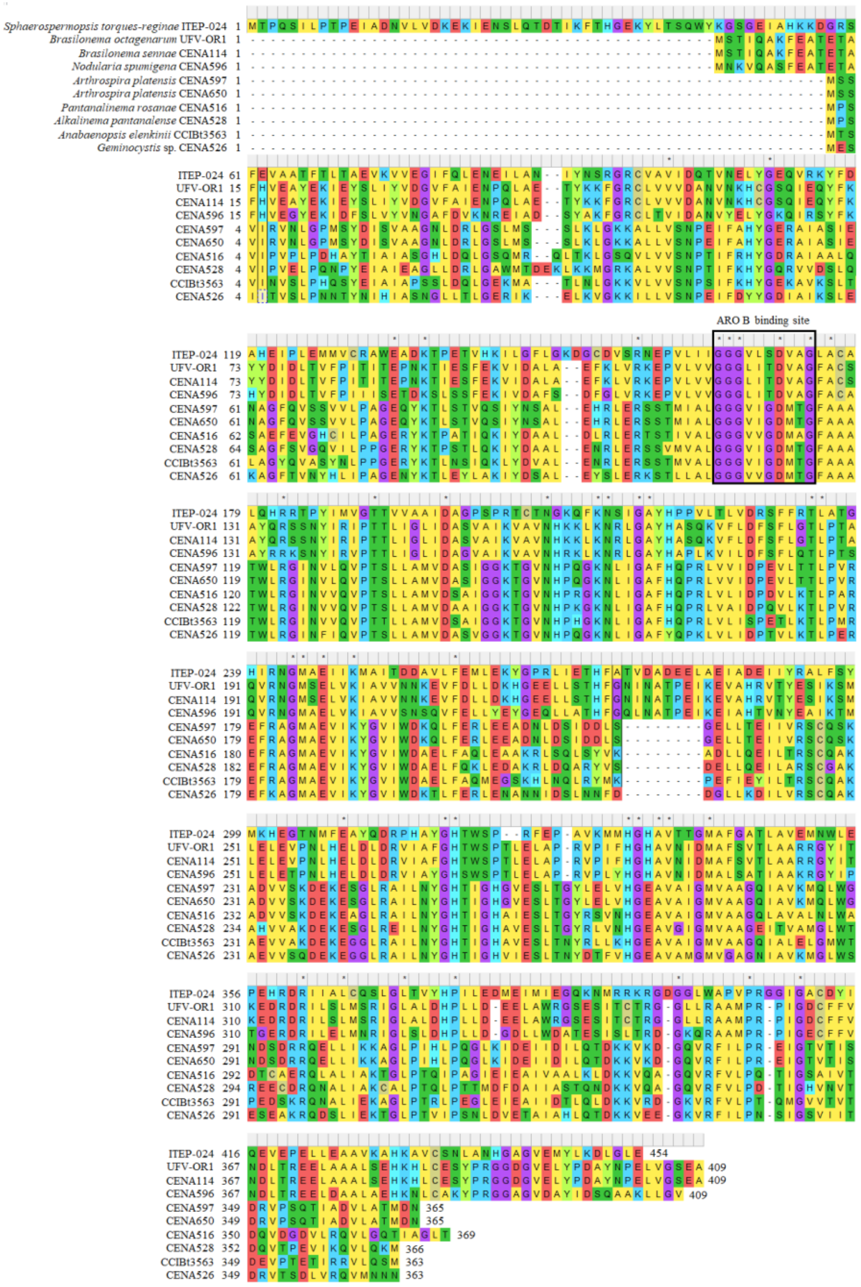

Figure S7. Comparison of the aligned deduced amino acid sequences of 3-dehydroquinate synthase from the ten strains used. Alignment performed in ClustalW. The (*) above each column represents the conserved amino acid positions, whereas the conservative substitutions are represented by the same colour scheme. The aroB binding site is boxed.

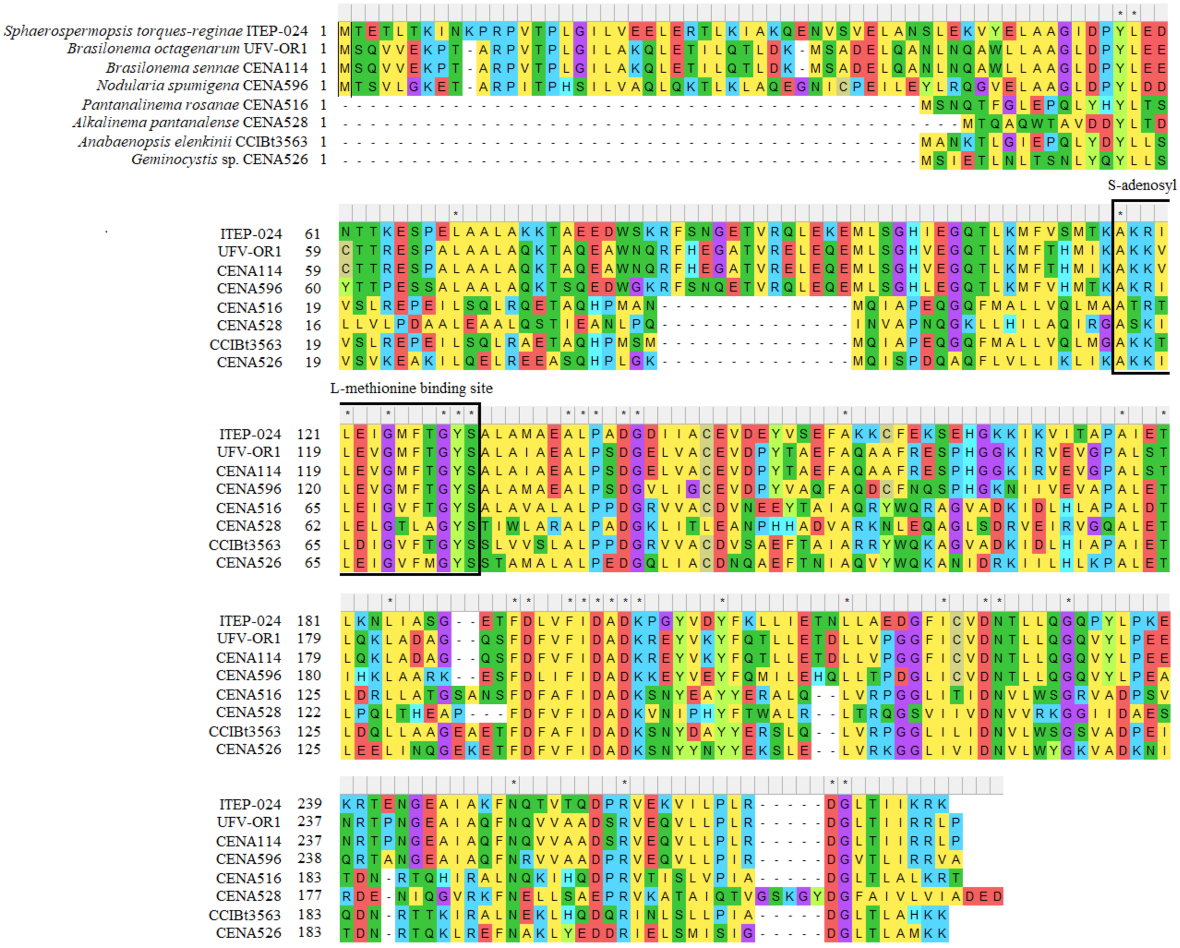

Figure S8. Comparison of the aligned deduced amino acid sequences of *O*-methyltransferase from the ten strains used. Alignment performed in ClustalW. The (*) above each column represents the conserved amino acid positions, whereas the conservative substitutions are represented by the same colour scheme. The S-adenosyl L-methionine binding site is boxed.

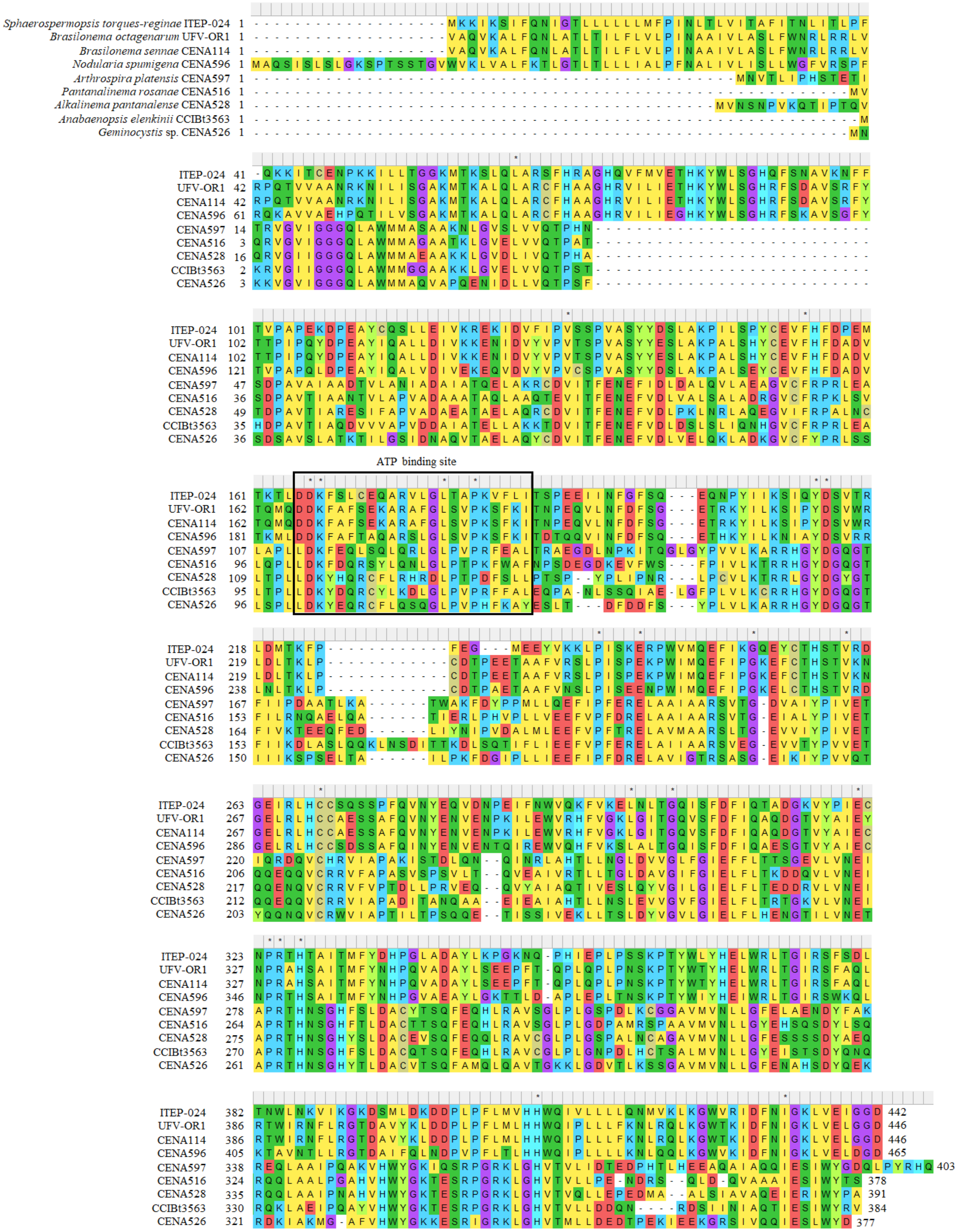

Figure S9. Comparison of the aligned deduced amino acid sequences of ATP-grasp from the ten strains used. Alignment performed in ClustalW. The (*) above each column represents the

conserved amino acid positions, whereas the conservative substitutions are represented by the same colour scheme. The ATP binding site is boxed.

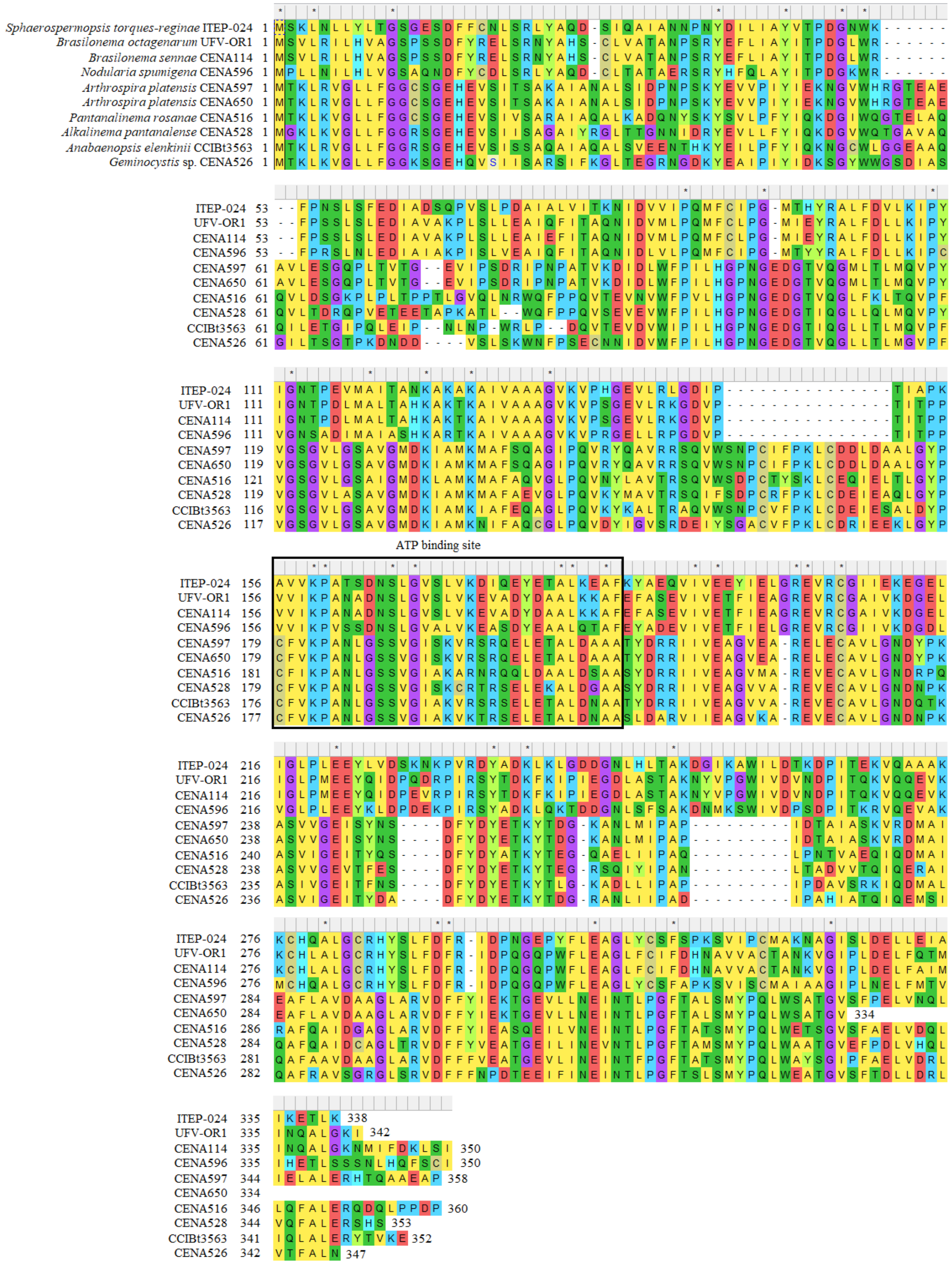

Figure S10. Comparison of the aligned deduced amino acid sequences of d-Ala d-Alanine ligase from the ten strains used. Alignment performed in ClustalW. The (*) above each column represents the conserved amino acid positions, whereas the conservative substitutions are represented by the same colour scheme. The ATP binding site is boxed.

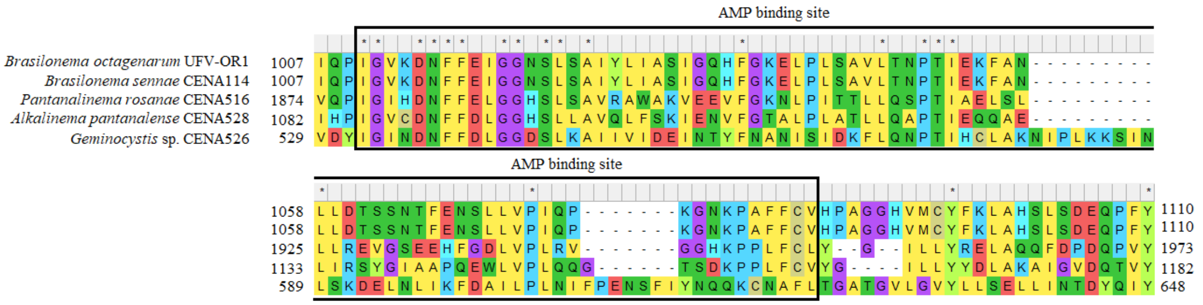

Figure S11. Comparison of the partial aligned deduced amino acid sequences of NRPS gene from the ten strains used, representing specifically the AMP binding site (boxed). Alignment performed in ClustalW. The (*) above each column represents the conserved amino acid positions, whereas the conservative substitutions are represented by the same colour scheme.

Table S1. Heat map based on the identity percentage of the protein encoded by the *mys*A gene annotated on each strain used in this study.

STRAINS	1	2	3	4	5	6	7	8	9	10
1.*Sphaerospermopsis torques-reginae* ITEP-024	100									
2.*Nodularia spumigena* CENA596	39.73	100								
3.*Anabaenopsis elenkinii* CCIBt3563	25.22	28.31	100							
4.*Arthrospira platensis* CENA597	29.67	28.41	66.12	100						
5.*Arthrospira platensis* CENA650	29.67	28.41	66.12	100	100					
6.*Geminocystis* sp. CENA526	28.14	29.48	61.33	65.56	65.56	100				
7.*Alkalinema pantanalense* CENA528	27.08	28.61	69.13	66.39	66.39	60.49	100			
8.*Pantanalinema rosanae* CENA516	28.01	24.65	69.25	64.54	64.54	62.64	73	100		
9.*Brasilonema sennae* CENA114	38.21	72.3	28.98	24.71	24.71	29.07	26.98	24.86	100	
10.*Brasilonema octagenarum* UFV-OR1	38.21	72.3	28.98	24.71	24.71	29.07	26.98	24.86	100	100

Table S2. Heat map based on the identity percentage of the protein encoded by the *mys*B gene annotated on each strain used in this study.

STRAINS	1	2	3	4	5	6	7	8
1.*Sphaerospermopsis torques-reginae* ITEP-024	100							
2.*Nodularia spumigena* CENA596	64.86	100						
3.*Anabaenopsis elenkinii* CCIBt3563	36.05	38.29	100					
4.*Geminocystis* sp. CENA526	39.55	35.06	60.91	100				
5.*Alkalinema pantanalense* CENA528	34.18	35.85	33.16	34.05	100			
6.*Pantanalinema rosanae* CENA516	39.89	40.53	78.64	56.82	33.86	100		
7.*Brasilonema sennae* CENA114	63.1	67.03	37.02	40.46	35.44	38.63	100	
8.*Brasilonema octagenarum* UFV-OR1	63.1	67.03	37.02	40.46	35.44	38.63	100	100

Table S3. Heat map based on the identity percentage of the protein encoded by the *mys*C gene annotated on each strain used in this study.

STRAINS	1	2	3	4	5	6	7	8	9
1.*Sphaerospermopsis torques-reginae* ITEP-024	100								
2.*Nodularia spumigena* CENA596	61.41	100							
3.*Anabaenopsis elenkinii* CCIBt3563	21.94	20.99	100						
4.*Arthrospira platensis* CENA597	19.92	22.32	63.92	100					
5.*Geminocystis* sp. CENA526	26.19	22.43	54.03	56.32	100				
6.*Alkalinema pantanalense* CENA528	0	0	60.42	58.22	53.72	100			
7.*Pantanalinema rosanae* CENA516	21.05	23.22	64.92	63.45	55.53	63.66	100		
8.*Brasilonema sennae* CENA114	60.31	75.28	0	21.21	23.53	0	0	100	
9.*Brasilonema octagenarum* UFV-OR1	60.09	75.06	0	21.21	23.53	0	0	99.78	100

Table S4. Heat map based on the identity percentage of the protein encoded by the *mys*D gene annotated on each strain used in this study.

STRAINS	1	2	3	4	5	6	7	8	9	10
1.*Sphaerospermopsis torques-reginae* ITEP-024	100									
2.*Nodularia spumigena* CENA596	64.6	100								
3.*Anabaenopsis elenkinii* CCIBt3563	23.5	22.68	100							
4.*Arthrospira platensis* CENA597	24.4	23.21	71.23	100						
5.*Arthrospira platensis* CENA650	24.02	23.21	71.47	100	100					
6.*Geminocystis* sp. CENA526	24.93	23.59	64.27	63.22	64.07	100				
7.*Alkalinema pantanalense* CENA528	23.37	21.3	69.14	66.38	66.57	65.04	100			
8.*Pantanalinema rosanae* CENA516	23.91	25.93	69.32	65.83	66.67	62.68	69.32	100		
9.*Brasilonema sennae* CENA114	61.65	67.54	23.63	24.58	24.58	22.67	23.62	26.19	100	
10.*Brasilonema octagenarum* UFV-OR1	61.36	68.53	22.01	24.58	24.58	23.99	21.69	26.79	98.53	100

Table S5. Motif analysis of each gene found on the evaluated strains. The numbers represent the e-value associated to each specific motif followed by their location within the gene in parentheses. (-) represents absence of significant hits for each motif.

Strain	3-dehydroquinate synthase (PF01761)	O-methyltransferases (PF01596)	ATP-grasp domain (PF02655 and PF02222)	d-Ala d-alanine ligase (PF07478)	Condensation domain+AMP-binding enzyme and its C-terminal +Phosphopantetheine binding site+Thioesterase domain (PF00668+PF00501+PF13193+PF00550 +PF00975)	Dioxygenases (PF02668)
**ITEP-024**	6.9 e^{-44} (128...385)	3.1 e^{-53} (89...276)	6.5 e^{-6} (179...309)	5.3 e^{-24} (156...334)	-	-
**CENA596**	2.3 e^{-55} (86...332)	7.0 e^{-54} (87...276)	9.1 e^{-5} (186...349)	9.8 e^{-19} (153...335)	-	-
**CENA114**	1.6 e^{-58} (83...331)	9.1 e^{-54} (87...275)	7.2 e^{-5} (240...330)	1.4 e^{-17} (152...335)	3.8 e^{-80} (10...470)/ 2.1 e^{-107} (491...884)/ 1.4 e^{-15} (892...966)/ 5.6 e^{-14} (996...1059)/ 3.2 e^{-30} (1084...1185)	7.7 e^{-22} (43...357)
**UFV-OR1**	1.6 e^{-58} (83...331)	8.8 e^{-53} (87...275)	0.0002 (240...330)	5.0 e^{-18} (152..335)	3.2 e^{-79} (10...470)/ 2.2 e^{-107} (491...884)/ 4.6 e^{-15} (892...966)/ 5.6 e^{-14} (996...1059)/ 1.1 e^{-30} (1084...1185)	9.3 e^{-22} (43...357)
**CENA597**	2.7 e^{-106} (69...329)	-	1.2 e^{-6} (112...281)	4.4 e^{-78} (139...345)	-	-
**CENA650**	2.7 e^{-106} (69...329)	-	-	4.1 e^{-73} (139...334)	-	-

**CCIBt3563**	9.1 e^{-107} (69...329)	4.4 e^{-78} (19...219)	4.6 e^{-59} (109...284)	2.2 e^{-76} (136...342)	-	-
**CENA528**	1.1 e^{-105} (72...332)	3.1 e^{-45} (24...203)	9.3 e^{-52} (127...289)	3.3 e^{-79} (140...345)	2.3 e^{-104} (76...527)/ 2.8 e^{-10} (702...956)/ 1.2 e^{-8} (964...1038)/ 6.9 e^{-13} (1071...1134)/ 1.0 e^{-18} (1202...1287)	-
**CENA516**	4.5 e^{-107} (70...330)	3.1 e^{-81} (19...219)	0.0012 (101...267)	1.4 e^{-77} (141...347)	9.8 e^{-111} (884...1327)/ *****6.0 e^{-101} (1384...1749)/ *****5.8 e^{-10} (1757...1832)/ *****2.8 e^{-13} (1862...1923)/ 2.1 e^{-19} (1958...2193)	-
**CENA526**	4.7 e^{-107} (70...329)	1.3 e^{-79} (19...219)	1.1 e^{-55} (110...275)	2.8 e^{-77} (137...343)	- / 7.2 e^{-85} (10...404)/ 1.2 e^{-7} (412...489)/ 6.0 e^{-11} (518...578)/ -	-

***CENA516** has a duplication on three motifs of NPRS-like complex (PF00501 in 289…686; PF13193 in 695…768; PF00550 in 800...862)

Table S6. Information concerning the encoding enzymes of the annotated genes of each strains used in this study.

Strain	Gene	Amino Acids (aa)	Proposed Function	Function of closest sequence	Most similar organism	Similarity (%)	Accession Number
**ITEP-024**	*mys*A	454	3-dehydroquinate synthase	3-dehydroquinate synthase	*Sphaerospermopsis reniformis*	96.92	QKX45747 (Geraldes et al. 2020)
**ITEP-024**	*mys*B	277	Methyltransferase	SAM-dependent methyltransferase	*Sphaerospermopsis kisseleviana*	93.50	QKX45748 (Geraldes et al. 2020)
**ITEP-024**	*mys*C	423	ATP-grasp ligase	ATP-grasp domain protein	*Sphaerospermopsis kisseleviana*	94.56	QKX45749 (Geraldes et al. 2020)
**ITEP-024**	*mys*D	340	d-Alanine ligase	d-Alanine d-Alanine ligase	*Sphaerospermopsis kisseleviana*	92.94	QKX45750 (Geraldes et al. 2020)
**CENA596**	*mys*A	409	3-dehydroquinate synthase	3-dehydroquinate synthase	*Nodularia spumigena* CCY9414	99.27	WP_063871916.1 (Popin et al. 2016)
**CENA596**	*mys*B	277	Methyltransferase	Caffeoyl-CoA *O*-methyltransferase	*Nodularia spumigena* CCY9414	97.47	WP_063871917.1 (Popin et al. 2016)
**CENA596**	*mys*C	465	ATP-grasp ligase	ATP-grasp domain protein	*Nodularia spumigena* CCY9414	92.47	WP_063871918.1 (Popin et al. 2016)

**CENA596**	*mys*D	350	d-Alanine ligase	d-Alanine d-Alanine ligase	*Nodularia spumigena* CCY9414	96.00	WP_063871919.1 (Popin et al. 2016)
**CENA597**	-	365	3-dehydroquinate synthase	3-dehydroquinate synthase	*Arthrospira platensis* YZ	95.34	-
**CENA597**	-	403	ATP-grasp ligase	Phosphoribosyl aminoimidazole carboxylase (PurK – ATP-grasp superfamily)	*Arthrospira platensis* YZ	100.00	-
**CENA597**	-	358	d-Alanine ligase	d-Alanine d-Alanine ligase	*Arthrospira platensis* YZ	100.00	-
**CENA650**	-	365	3-dehydroquinate synthase	3-dehydroquinate synthase	*Arthrospira platensis* YZ	95.34	-
**CENA650**	-	334	d-Alanine ligase	d-Alanine d-Alanine ligase	*Arthrospira platensis* YZ	100.00	-
**CENA516**	-	369	3-dehydroquinate synthase	3-dehydroquinate synthase	*Nostoc* sp. TCL240-02	76.73	-
**CENA516**	-	221	Methyltransferase	SAM-dependent methyltransferase	*Tolypothrix* sp. PCC 7910	80.37	-
**CENA516**	-	378	ATP-grasp ligase	phosphoribosyl aminoimidazole carboxylase (PurK – ATP-grasp superfamily)	*Nostoc* sp. NIES-2111	63.42	-
**CENA516**	-	360	d-Alanine ligase	d-Alanine d-Alanine ligase	*Calothrix brevissima* NIES-22	71.31	-
**CENA516**	-	2202	NRPS-like	Non-ribosomal peptide synthase/polyketide synthase	*Nostoc* sp. UHCC 0702	45.21	-
**CENA528**	-	366	3-dehydroquinate synthase	3-dehydroquinate synthase (AroB)	*Leptolyngbya* sp. NIES-3755	73.91	-
**CENA528**	-	223	Methyltransferase	*O*-methyltransferase family protein	*Calothrix* sp. NIES-4101	79.73	-

**CENA528**	-	391	ATP-grasp ligase	5-(carboxyamino)imidazole ribonucleotide synthase (ATP-grasp superfamily)	*Nostoc* sp. ATCC 53789	62.27	-
**CENA528**	-	353	d-Alanine ligase	d-Alanine d-Alanine ligase	*Crocosphaera subtropica* ATCC 51142	75.14	-
**CENA528**	-	1415	NRPS-like	Non-ribosomal peptide synthetase	Nostoc sp. CENA543	44.32	-
**CCIBt3594**	-	363	3-dehydroquinate synthase	3-dehydroquinate synthase	*Nodularia spumigena* CCY9414	87.10	-
**CCIBt3594**	-	220	Methyltransferase	*O*-methyltransferase	*Nodularia spumigena* CCY9414	91.36	-
**CCIBt3594**	-	384	ATP-grasp ligase	N5-carboxyaminoimidazole ribonucleotide synthase (ATP-grasp superfamily)	*Nodularia sphaerocarpa* UHCC 0038	84.29	-
**CCIBt3594**	-	352	d-Alanine ligase	d-Alanine d-Alanine ligase	*Nodularia spumigena* CCY9414	86.65	-
**CENA526**	-	363	3-dehydroquinate synthase	3-dehydroquinate synthase	*Geminocystis* sp. NIES-3709	80.56	-
**CENA526**	-	220	Methyltransferase	*O*-methyltransferase	*Geminocystis* sp. NIES-3709	76.36	-
**CENA526**	-	377	ATP-grasp ligase	Phosphoribosyl aminoimidazole carboxylase (PurK – ATP-grasp superfamily)	*Geminocystis* sp. NIES-3709	81.43	-
**CENA526**	-	347	d-Alanine ligase	d-Alanine d-Alanine ligase	*Geminocystis* sp. NIES-3709	84.15	-

**CENA526**	-	973	NRPS-like	Malonyl CoA-acyl carrier protein	*Geminocystis* sp. NIES-3709	71.44	-
**CENA114**	*mys*A	411	3-dehydroquinate synthase	3-dehydroquinate synthase	*Brasilonema octagenarum* UFV-E1	100.00	CP030118.1 (Alvarenga et al. 2020)
**CENA114**	*mys*B	276	Methyltransferase	SAM-dependent methyltransferase	*Brasilonema octagenarum* UFV-E1	95.29	CP030118.1 (Alvarenga et al. 2020)
**CENA114**	*mys*C	446	ATP-grasp ligase	ATP-grasp enzyme	*Brasilonema octagenarum* UFV-E1	100.00	CP030118.1 (Alvarenga et al. 2020)
**CENA114**	*mys*D	350	d-Alanine ligase	d-Alanine d-Alanine ligase	*Brasilonema octagenarum* UFV-E1	97.14	CP030118.1 (Alvarenga et al. 2020)
**CENA114**	*mys*E	1340	NRPS-like	Amino acid adenylation domain protein	*Brasilonema octagenarum* UFV-E1	97.24	CP030118.1 (Alvarenga et al. 2020)
**CENA114**	*mys*H	387	Dioxygenase	TfdA family catabolism dioxygenase	*Brasilonema octagenarum* UFV-E1	99.91	CP030118.1 (Alvarenga et al. 2020)
**UFV-OR1**	*mys*A	411	3-dehydroquinate synthase	3-dehydroquinate synthase	*Brasilonema octagenarum* UFV-E1	100.00	QMEC01000014.1 (Alvarenga et al. 2020)
**UFV-OR1**	*mys*B	276	Methyltransferase	SAM-dependent methyltransferase	*Brasilonema octagenarum* UFV-E1	95.29	QMEC01000014.1 (Alvarenga et al. 2020)
**UFV-OR1**	*mys*C	446	ATP-grasp ligase	ATP-grasp enzyme	*Brasilonema octagenarum* UFV-E1	99.78	QMEC01000014.1 (Alvarenga et al. 2020)
**UFV-OR1**	*mys*D	342	d-Alanine ligase	d-Alanine d-Alanine ligase	*Brasilonema octagenarum* UFV-E1	95.60	QMEC01000014.1 (Alvarenga et al. 2020)
**UFV-OR1**	*mys*E	1340	NRPS-like	Amino acid adenylation domain protein	*Brasilonema octagenarum* UFV-E1	96.64	QMEC01000014.1 (Alvarenga et al. 2020)

**UFV-OR1**	*mys*H	387	Dioxygenase	TfdA family catabolism dioxygenase	*Brasilonema octagenarum* UFV-E1	99.91	QMEC01000014.1 (Alvarenga et al. 2020)

Table S7. Concentration of total carotenoids calculated in each cyanobacterial strain according to experimental group (fluorescent light and UV-exposed) presented in mg L^{-1}. (*) indicates significant statistical difference between conditions by ANOVA test ($p < 0.005$)

Strain	Day 45 (control/without UV)	Day 45 (72h UV-exposure)
***Sphaerospermopsis torques-reginae* ITEP-024**	0.71 ± 0.01*	0.30 ± 0.10*
***Nodularia spumigena* CENA596**	0.56 ± 0.02*	0.22 ± 0.04*
***Brasilonema sennae* CENA114**	0.53 ± 0.09	0.52 ± 0.002
***Brasilonema octagenarum* UFV-OR1**	0.51 ± 0.03	0.52 ± 0.04
***Arthrospira platensis* CENA597**	0.43 ± 0.02*	0.28 ± 0.04*
***Arthrospira platensis* CENA650**	0.42 ± 0.04*	0.35 ± 0.01*
***Anabaenopsis elenkinii* CCIBt3563**	0.79 ± 0.08	0.71 ± 0.06
***Alkalinema pantanalense* CENA528**	0.79 ± 0.08	0.85 ± 0.07
***Pantanalinema rosanae* CENA516**	0.86 ± 0.06	0.72 ± 0.08
***Geminocystis* sp. CENA526**	0.52 ± 0.02*	0.70 ± 0.03*

Table S8. Culture media nutritional comparison.

Component	Z8 (g L^{-1})	BG-11 (g L^{-1})	ASM-1 (g L^{-1})
$NaNO_3$	4.6 x10^{-1}	1.5	1.0 x10^{-2}
$Ca(NO_3)_2$	6.0 x10^{-2}	-	-
$CaCl_2$	-	3.6 x10^{-2}	3.0 x10^{-5}
$MgSO_4$	2.5 x10^{-2}	7.5 x10^{-2}	5.0 x10^{-5}
K_2HPO_4	3.1 x10^{-2}	4.0 x10^{-2}	1.7 x10^{-5}
Na_2CO_3	2.1 x10^{-2}	2.0 x10^{-2}	-
Na_2HPO_4	-	-	2.7 x10^{-5}
EDTA Na_2	4.3 x10^{-3}	1.0 x10^{-3}	7.4 x10^{-6}
$FeCl_3$	3.1 x10^{-3}	-	1.1 x10^{-6}
$(NH_4)_5[Fe(C_6H_4O_7)_2]$	-	6.0 x10^{-3}	-
$C_6H_8O_7$	-	6.0 x10^{-3}	-
$CoNO_3$	1.5 x10^{-5}	5.0 x10^{-5}	-
$CoCl_2$	-	-	2.0 x10^{-8}
$CuSO_4$	1.2 x10^{-5}	8.0 x10^{-5}	-
$CuCl_2$	-	-	1.4 x10^{-9}
$MnSO_4$	1.3 x10^{-3}	-	-
$MnCl_2$	-	1.8 x10^{-3}	1.4 x10^{-6}
H_3BO_3	3.1 x10^{-3}	-	2.5 x10^{-6}
H_2BO_4	-	2.9 x10^{-3}	-
$ZnSO_4$	2.9 x10^{-5}	2.2 x10^{-4}	-
$ZnCl_2$	-	-	3.4 x10^{-7}
KBr	1.2 x10^{-5}	-	-
KI	8.3 x10^{-6}	-	-
$CdNO_3$	1.5 x10^{-5}	-	-
$NiSO_4$	2.0 x10^{-5}	-	-
$CrNO_3$	4.1 x10^{-5}	-	-

(-) indicates that the specific component is absent in the culture media
